# Naringenin and Phytoestrogen 8-Prenylnaringenin Protect against Islet Dysfunction and Inhibit Apoptotic Signaling in Insulin-Deficient Diabetic Mice

**DOI:** 10.3390/molecules27134227

**Published:** 2022-06-30

**Authors:** Song Park, Kyu-Sang Sim, Yeop Hwangbo, Sung-Jin Park, Young-Jun Kim, Jun-Ho Kim

**Affiliations:** 1 Department of Food Science and Biotechnology, Andong National University, Andong 36729, Korea; andongori@naver.com (S.P.); qhduq97@naver.com (Y.H.); 2 Biomaterials Research Institute, Kyochon F&B, Andong 36729, Korea; sim9612@naver.com; 3 Department of Food and Biotechnology, Korea University, Sejong 30019, Korea; timothypsj@hanmail.net (S.-J.P.); yk46@korea.ac.kr (Y.-J.K.)

**Keywords:** naringenin, 8-prenylnaringenin, diabetes, islet dysfunction, β-cell apoptosis, estrogen receptor-α

## Abstract

It has been shown that citrus flavanone naringenin and its prenyl derivative 8-prenylnaringenin (8-PN) possess various pharmacological activities in in vitro and in vivo models. Interestingly, it has been proposed that prenylation can enhance biological potentials, including the estrogen-like activities of flavonoids. The objective of this study was to investigate the anti-diabetic potential and molecular mechanism of 8-PN in streptozotocin (STZ)-induced insulin-deficient diabetic mice in comparison with naringenin reported to exhibit hypoglycemic effects. The oral administration of naringenin and 8-PN ameliorated impaired glucose homeostasis and islet dysfunction induced by STZ treatment. These protective effects were associated with the suppression of pancreatic β-cell apoptosis and inflammatory responses in mice. Moreover, both naringenin and 8-PN normalized STZ-induced insulin-signaling defects in skeletal muscles and apoptotic protein expression in the liver. Importantly, 8-PN increased the protein expression levels of estrogen receptor-α (ERα) in the pancreas and liver and of fibroblast growth factor 21 in the liver, suggesting that 8-PN could act as an ERα agonist in the regulation of glucose homeostasis. This study provides novel insights into the mechanisms underlying preventive effects of naringenin and 8-PN on the impairment of glucose homeostasis in insulin-deficient diabetic mice.

## 1. Introduction

Naringenin (4,5,7-trihydroxy-flavanone), a predominant flavanone in citrus fruits, has drawn growing scientific attention due to its bioavailability and pharmacological properties including antioxidant, anti-inflammatory, anti-atherosclerotic, anticancer, antivirus, and hepatoprotective activities [1,2]. One of the most notable bioactivities of naringenin is its anti-diabetic potential, which has been demonstrated in both in vitro and in vivo studies. In pancreatic β-cells, naringenin improved glucose-stimulated insulin secretion and reduced pro-apoptotic mRNA levels [3]. More importantly, oral administration of naringenin for 3 to 16 weeks ameliorated hyperglycemia, oxidative stress, inflammatory response, and hepatotoxicity in type 1 and/or type 2 diabetic animal models [4,5,6,7]. In addition to these anti-diabetic activities of naringenin, we have also demonstrated that naringin, a glycoside form of naringenin, could protect mice against pancreatic islet dysfunction by inhibiting oxidative stress-induced β-cell apoptosis [8]. These findings suggest that naringin and naringenin as citrus flavonoids could be used as promising anti-diabetic alternatives. However, to fully understand their regulatory effects on glucose homeostasis, more molecular studies are needed to investigate their tissue-specific effects.

With increasing scientific evidence regarding pharmacological effects of naringenin, 8-prenylnaringenin (8-PN), a prenyl derivative of naringenin, has also been in the spotlight recently as a natural therapeutic agent. It is now believed that prenylation can enhance biological activities, such as the antioxidant, anti-inflammatory, and antibacterial properties of flavonoids [9]. In fact, in comparison with naringenin, 8-PN has shown more potent anticancer activity [10], bone formation effect [11], and activity preventing disuse muscle atrophy [12]. Of note, 8-PN has been considered as a novel therapeutic for treating post-menopausal symptoms [13,14]. In our recent comparative study of phytoestrogens, 8-PN and coumestrol exhibited the most potent estrogen receptor (ER) agonistic activity in both in vitro and in vivo assays [15]. Although increasing evidence has indicated the potential of prenylated flavanone 8-PN as a promising therapeutic agent, there is currently limited information on its anti-diabetic properties. Luis et al. [16] reported that 8-PN downregulates galectin-3 overexpression, an oxidative biomarker, in the liver and kidney of type 2 diabetic mice induced by high-fat diet feeding. Using the same mouse model, their research group has also found that 8-PN treatment activates AMP-activated protein kinase and reduces lipogenic enzyme expression in the liver and muscle [17]. However, the protective role of 8-PN against pancreatic β-cell failure has not been elucidated yet. Because β-cell death or dysfunction is the critical precipitating event in the development of both type 1 and type 2 diabetes, the objective of this study was to investigate protective effects of 8-PN in comparison with those of naringenin against pancreatic β-cell failure and related tissue damages in streptozotocin (STZ)-induced insulin-deficient diabetic mice. STZ is taken up by pancreatic β-cells via glucose transporter 2 and causes β-cell death via multiple mechanisms including DNA methylation, nitric oxide production, and the generation of reactive oxygen species [18]. It has also been demonstrated that STZ promotes leukocyte infiltration into pancreatic islets and, subsequently, induces cytokine-mediated apoptosis in β-cells [8]. Therefore, in this study, we focused on the inhibitory effects of naringenin and 8-PN on STZ-induced inflammatory response and apoptosis signaling.

## 2. Results

### 2.1. Naringenin and 8-PN Ameliorate Impaired Glucose Homeostasis and Islet Dysfunction in Mice Exposed to STZ

**Figure 1:**
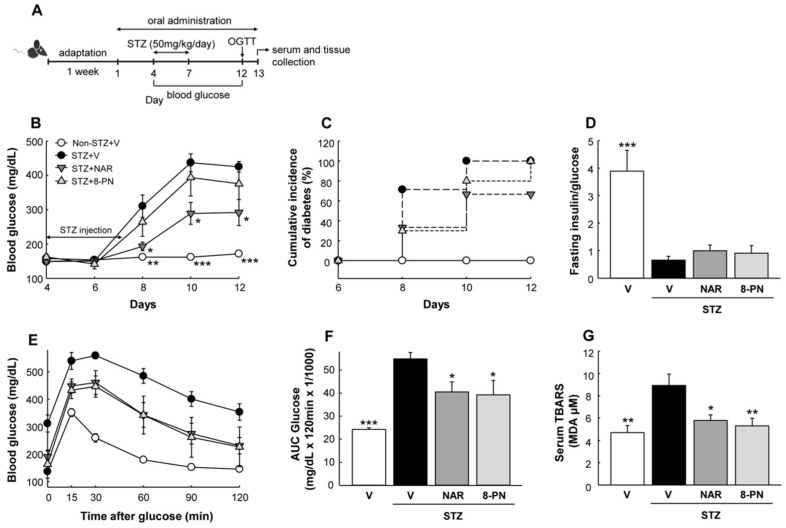
Effects of naringenin and 8-PN on glucose homeostasis. (**A**) Timeline of the study. On the 4th day of oral administration, diabetes was induced by intraperitoneal injection of STZ at a dose of 50 mg/kg/day for four consecutive days. (**B**) Random-fed blood glucose concentrations. (**C**) Cumulative incidence of diabetes was calculated as the percentage of hyperglycemic mice (glucose level ≥ 250 mg/dL) at each time point. (**D**) The ratio of fasting serum insulin (pg/mL) to blood glucose (mg/dL) was used as an index of insulin deficiency in mi€ (**E**) Glucose concentrations during glucose tolerance testing with (**F**) corresponding area under the curve (AUC) (×1/1000) were measured at Day 12. (**G**) Thiobarbituric acid reactive substances (TBARS) concentrations in serum were expressed in terms of malondialdehyde (MDA) equivalents. V, vehicle; NAR, naringenin; 8-PN, 8-prenylnaringenin. Data are shown as means ± SEM (n = 6). * *p* < 0.05, ** *p* < 0.01, *** *p* < 0.001 vs. STZ-vehicle group.

Figure 1A shows a timeline of the animal experiment. Diabetes was induced by multiple-low-dose STZ treatment for four consecutive days. The administration of naringenin at 50 mg/kg suppressed progressive hyperglycemia and diabetes incidence (blood glucose ≥ 250 mg/dL) induced by STZ (Figure 1B,C). Compared to naringenin, 8-PN showed a weaker hypoglycemic effect on mice. The fasting insulin-to-glucose ratio, an index of insulin resistance [19], was slightly but not significantly increased by both naringenin and 8-PN compared to that in the STZ control group (Figure 1D). Following an oral glucose load, both naringenin and 8-PN improved glucose tolerance at a similar level in STZ-treated mice (Figure 1E,F). Moreover, the serum levels of thiobarbituric acid reactive substances (TBARS) as an indicator of oxidative stress were significantly decreased by both naringenin and 8-PN (Figure 1G).

**Figure 2:**
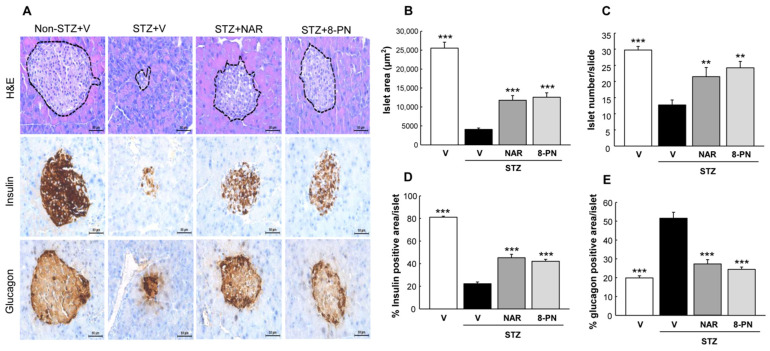
Protective effects of naringenin and 8-PN against islet dysfunction. (**A**) H&E and DAB staining for insulin and glucagon were performed to analyze histopathological changes in pancreatic islets (×400) at the end of study. H&E-stained sections were analyzed for the measurement of (**B**) islet area and (**C**) islet number using ImageJ software. Quantitative data of (**D**) insulin and (**E**) glucagon signals were calculated by dividing the area of target signal by the total islet area, for at least 10 islets per mouse. V, vehicle; NAR, naringenin; 8-PN, 8-prenylnaringenin. Data are shown as means ± SEM (n = 4). ** *p* < 0.01, *** *p* < 0.001 vs. STZ vehicle group.

Consistent with changes of blood glucose, STZ-treated mice showed pancreatic islet deterioration and dysfunction, as reflected by reduced size and number of islets and impaired signal distribution of insulin and glucagon (Figure 2). However, naringenin and 8-PN protected mice against STZ-induced islet dysfunction. The effects of 8-PN were comparable to those of naringenin.

### 2.2. Naringenin and 8-PN Suppress STZ-Induced Pancreatic β-Cell Apoptosis

**Figure 3:**
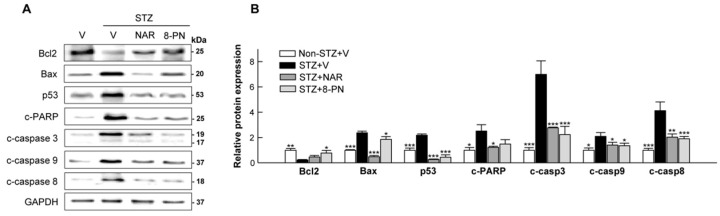
Inhibitory effects of naringenin and 8-PN on apoptotic signaling in the pancreas. (**A**) Representative Western blot images of pancreatic Bcl2, Bax, p53, cleaved PARP, cleaved caspase-3, cleaved caspase-9, and cleaved caspase-8 and (**B**) corresponding quantitative data were used for evaluating β-cell apoptosis. V, vehicle; NAR, naringenin; 8-PN, 8-prenylnaringenin. Data are shown as means ± SEM (n = 6). * *p* < 0.05, ** *p* < 0.01, *** *p* < 0.001 vs. STZ-vehicle group.

It is well known that multiple low-dose STZ treatments can induce pancreatic β-cell apoptosis that is central to the pathogenesis of type 1 and type 2 diabetes [20]. To examine whether naringenin and 8-PN could protect mice against STZ-induced islet dysfunction by suppressing apoptotic β-cell death, we analyzed the expression levels of major proteins involved in intrinsic and extrinsic apoptosis pathways. As expected, STZ-vehicle mice showed significantly increased expression levels of apoptotic proteins, including cleaved caspase-3, -9, and -8 (executors of intrinsic and extrinsic apoptosis) in the pancreas (Figure 3). However, such apoptotic response in STZ-treated mice was reversed by naringenin and 8-PN treatments, indicating their protective effects against STZ-induced β-cell apoptosis.

### 2.3. Naringenin and 8-PN Inhibit STZ-Induced Inflammatory Responses in Pancreas

**Figure 4:**
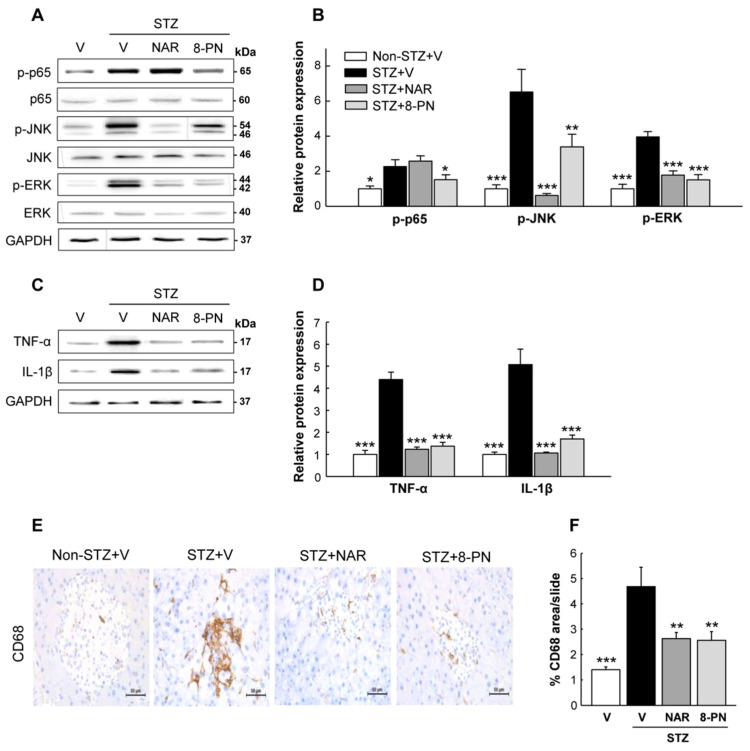
Inhibitory effects of naringenin and 8-PN on macrophage infiltration and inflammatory signaling in the pancreas. (**A**) Representative Western blot images of phospho-p65, p65, phospho-JNK, JNK, phospho-ERK, and ERK in the pancreas and (**B**) corresponding quantitative data. (**C**) Representative Western blot images of TNF-α and IL-1β in the pancreas and (**D**) corresponding quantitative data. (**E**) Pancreas DAB staining for CD68^+^ macrophages (×400). (**F**) Quantitative data of macrophage infiltration were calculated by dividing the area of target signal by the total area. Data are shown as means ± SEM (n = 6 for (**A**–**D**) and n = 4 for (**E**,**F**)). * *p* < 0.05, ** *p* < 0.01, *** *p* < 0.001 vs. STZ-vehicle group.

To investigate the possible upstream signaling involved in the suppression of β-cell apoptosis by naringenin and 8-PN, we monitored the changes of inflammatory responses in the pancreas next. Along with markedly increased expression levels of TNF-α and IL-1β proteins, the phosphorylation levels of p65, JNK, and ERK as key mediators of cytokine-induced β-cell apoptosis [21] were enhanced in STZ-vehicle mice (Figure 4A–D). However, these STZ-induced changes were effectively inhibited by naringenin and 8-PN except for the expression of phosphorylated p65 in the STZ + naringenin group. Moreover, histological examination showed marked peri- and intra-islet infiltration of CD68^+^ macrophages in the STZ-vehicle group, which was significantly reduced by naringenin and 8-PN treatments (Figure 4E,F). These findings suggest that both naringenin and 8-PN could counteract STZ-induced β-cell apoptosis by inhibiting macrophage infiltration into the pancreas and subsequent cytokine-induced NF-κB and MAPK activations.

### 2.4. Naringenin and 8-PN Counteract STZ-Induced Insulin-Signaling Defect in Skeletal Muscle and Hepatic Apoptosis

**Figure 5:**
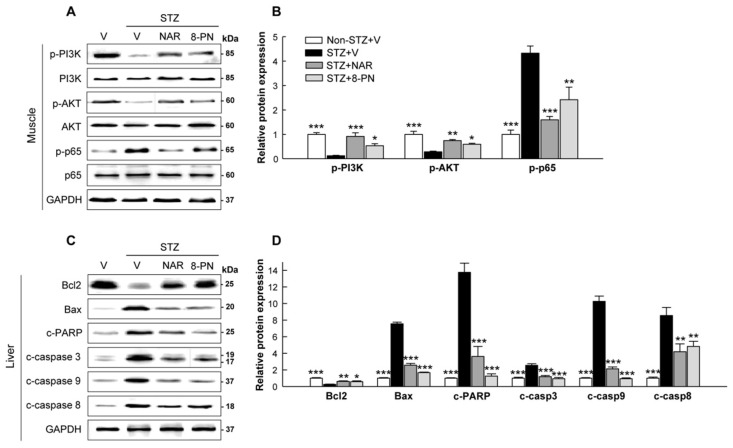
Effects of naringenin and 8-PN on insulin-signaling defect in skeletal muscle and hepatic apoptosis. (**A**) Representative Western blot images of phospho-PI3K, PI3K, phospho-AKT, AKT, phospho-p65, and p65 in the skeletal muscle and (**B**) corresponding quantitative data. (**C**) Representative Western blot images of Bcl2, Bax, cleaved PARP, cleaved caspase-3, cleaved caspase-9, and cleaved caspase-8 in the liver and (**D**) corresponding quantitative data. V, vehicle; NAR, naringenin; 8-PN, 8-prenylnaringenin. Data are shown as means ± SEM (n = 6). * *p* < 0.05, ** *p* < 0.01, *** *p* < 0.001 vs. STZ vehicle group.

In addition to the pancreas, the skeletal muscle and liver are also considered as target tissues in which STZ can directly or indirectly show its cytotoxic effects [22,23]. The PI3K/Akt pathway has been identified as one of the most important pathways for insulin-stimulated glucose uptake in skeletal muscles [24]. Moreover, NF-κB activation is thought to play a key role in glucose metabolism abnormalities and muscle wasting [25]. In this study, STZ treatment markedly reduced the expression levels of phosphorylated PI3K and AKT, whereas it increased p65 phosphorylation in skeletal muscles (Figure 5A,B), indicating insulin-signaling defects by STZ. Importantly, naringenin and 8-PN significantly reversed the expression levels of these muscle proteins induced by STZ. In addition, consistent with their inhibitory effects on pancreatic apoptosis, their treatments normalized apoptotic protein expressions in the liver, which were significantly increased by STZ (Figure 5C,D).

### 2.5. Changes of Estrogen Receptors and FGF21 Protein Expression in Target Tissues

**Figure 6:**
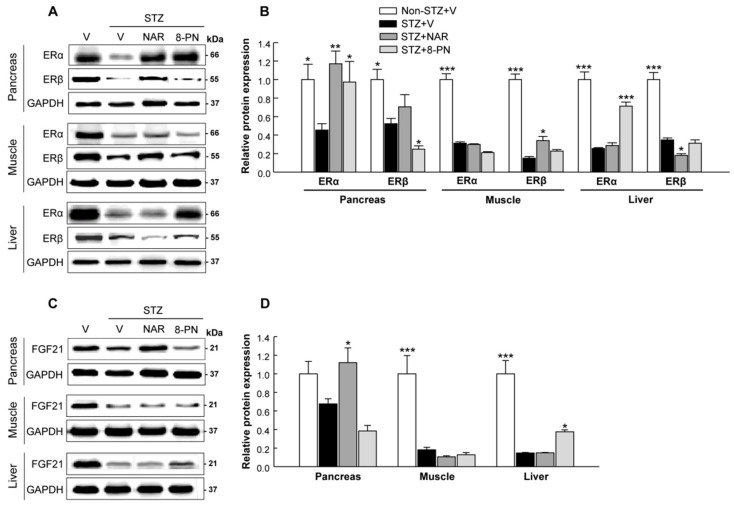
Effects of naringenin and 8-PN on protein expressions of estrogen receptors and FGF21 in target tissues. (**A**) Representative Western blot images of estrogen receptor-α and -β in the pancreas, skeletal muscle, and liver and (**B**) corresponding quantitative data. (**C**) Representative Western blot images of fibroblast growth factor 21 in the pancreas, skeletal muscle, and liver and (**D**) corresponding quantitative data. V, vehicle; NAR, naringenin; 8-PN, 8-prenylnaringenin. Data are shown as means ± SEM (n = 6). * *p* < 0.05, ** *p* < 0.01, *** *p* < 0.001 vs. STZ-vehicle group.

Because 8-PN has been classified as a potent phytoestrogen that is structurally similar to estrogens with ER-agonistic and/or -antagonistic activity, we examined changes in ERα and ERβ protein expressions. Interestingly, in the pancreas, skeletal muscle, and liver of STZ-vehicle mice, both ERα and ERβ protein expression levels were significantly reduced compared to those of normal control mice (Figure 6A,B). Naringenin and 8-PN differentially regulated ERα and ERβ protein expression levels according to target tissues. Of note, in STZ-treated mice, ERα expression was normalized by naringenin and 8-PN in the pancreas and by 8-PN in the liver. Additionally, the protein expression of fibroblast growth factor 21 (FGF21)—a key regulator of glucose metabolism—was reduced by STZ but increased by naringenin in the pancreas and by 8-PN in the liver (Figure 6C,D).

## 3. Discussion

Naringin and naringenin as citrus flavonoids have been shown to display strong hypoglycemic effects with the ability to protect β-cell functions, suggesting their potential as promising anti-diabetic alternatives [5,8]. The most important finding of this study was that 8-PN, a prenyl derivative of naringenin, could protect the pancreas from STZ-induced β-cell apoptosis and inflammatory responses. Its effects were comparable to those of naringenin. In autoimmune diabetes, the migration of macrophages to pancreatic islets and the subsequent production of inflammatory cytokines are critical events that can lead to β-cell death and islet deterioration [21]. Cytokine-induced NF-κB-/MAPK-mediated pathways have been shown to activate both intrinsic and extrinsic apoptosis in β-cells [21,26]. In the present study, naringenin and 8-PN inhibited the expression levels of pro-apoptotic proteins involved in both intrinsic and extrinsic pathways (Figure 3). These effects were associated with the suppression of macrophage infiltration into the pancreas and inflammatory responses induced by STZ (Figure 4). Moreover, both naringenin and 8-PN protected skeletal muscles against STZ-induced insulin-signaling defects, as shown by the normalization of PI3K, AKT, and p65 phosphorylation (Figure 5). In cellular responses to insulin, cytokines, and many other growth factors, PI3K phosphorylates AKT, which then activates downstream regulators involved in many cellular processes such as apoptosis, metabolism, and cell cycle progression [27,28]. Insulin controls skeletal muscle metabolism by promoting glucose uptake and glycogen synthesis via the PI3K/AKT signaling pathway. On the other hand, activated NF-κB signaling is closely associated with the pathogenesis of insulin resistance [29] as well as muscle loss and weakness [30]. Therefore, current results suggest similar action mechanisms of naringenin and 8-PN in preventing pancreatic islet dysfunction and muscle insulin resistance, which could explain their anti-diabetic potential.

Because 8-PN has been identified as a phytoestrogen that has potent ER-agonistic activity [31], the question of whether 8-PN displays its pharmacological effects in an ER-dependent manner or not is an important issue for understanding its molecular mechanism. In contrast to most other phytoestrogens that show higher binding affinity for ERβ than for ERα, 8-PN has been shown to have a higher affinity for ERα in in vitro competitive binding assays and transfected yeast cells [32,33]. This preferential binding activity of 8-PN for ERα is believed to enable its pharmacological roles, particularly in the regulation of bone and energy metabolism [14,32]. In this study, STZ treatment markedly reduced the protein expression levels of both Erα and Erβ in the pancreas, skeletal muscle, and liver (Figure 6). This result is consistent with previous findings, showing reduced expressions of ERα and ERβ by STZ in kidney and brain tissues [34,35]. Importantly, the expression levels of ERα, but not ERβ, were reversed by 8-PN in the pancreas and liver of STZ-treated mice. It has been well established that ERα plays an important role in β-cell survival and islet function [36,37]. Le May et al. [37] reported that 17β-estradiol suppresses apoptosis and promotes insulin secretion in the islets of wild-type mice. However, its protection is partially abolished in the islets of Erα-knockout mice. In addition, it has been demonstrated that hepatic ERα activation can enhance energy expenditure with an increased FGF21 expression in the liver of mice [38]. Consistently, in this study, 8-PN increased the hepatic protein expression of FGF21, which was dramatically reduced by STZ treatment (Figure 6). FGF21 is primarily produced in the liver and acts as a central metabolic regulator, which promotes glucose uptake and suppresses glucose release from the liver [39]. Thus, our results indicate that ERα activation could be associated with protective effects of 8-PN observed in the pancreas and liver. However, further studies using ER-knockout rodent models and/or ER antagonists are needed to clearly elucidate the estrogen-like action of 8-PN in the regulation of glucose homeostasis. 

Hop (*Humulus lupulus* L.) is a major source of prenylated flavonoids that possess a prenyl group attached to the flavane nucleus. In hops, prenylated flavonoids are divided into prenylated chalcones (xanthohumol and desmethylxanthohumol) and prenylated flavanones (6-prenylnaringenin and 8-PN) according to their structural distinction [14]. In addition to 8-PN as a neutraceutical agent, recent studies have demonstrated the potential of 6-prenylnaringenin as a sleep modulator [40] and an anti-melanoma agent [41]. However, the industrial application of these prenylated flavanones has been limited due to their very low concentrations in hops and beer [42]. Against this backdrop, several attempts have recently been made to achieve a maximum yield of 8-PN in hop materials [13] or to develop an efficient synthetic method of 6-prenylnaringenin and 8-PN from xanthohumol [42]. In fact, through the magnesium oxide-catalyzed thermal isomerization of hop materials, the yield of 8-PN increased up to 70% relative to the starting material [13]. In addition, Schaefer et al. [43] suggested the use of a radioimmunoassay for the quantitative determination of 8-PN in biological matrices. Therefore, along with continuing exploration of the novel biological activity of prenylated flavonoids, these studies will help to expand their industrial application as a nutraceutical and/or therapeutic agent.

Taken together, our study showed that naringenin and 8-PN could protect mice against STZ-induced pancreatic islet dysfunction by inhibiting pro-inflammatory cascade and β-cell apoptosis. In addition, naringenin and 8-PN counteracted the impairment of insulin signaling in skeletal muscle and hepatic apoptosis. Increased ERα protein expressions in the pancreas and liver by 8-PN suggest its possible action mechanism as an ERα agonist in the regulation of glucose homeostasis. These findings provide novel insights into the mechanisms underlying anti-diabetic effects of naringenin and 8-PN and suggest further clinical studies.

## 4. Materials and Methods

### 4.1. Animal Study

Seven-week-old male C57BL/6J mice (Orient Bio Inc., Seongnam-Si, Korea) were housed under controlled temperature and humidity with 12 h light-dark cycles. After an acclimation period of one week, mice were randomly divided into four treatment groups as follows: (1) non-STZ + vehicle, (2) STZ + vehicle, (3) STZ + naringenin, and (4) STZ + 8-PN. Naringenin and 8-PN (ChemFaces Biochemical, Wuhan, China) were dissolved in saline containing 2% Tween-80 and 0.5% methylcellulose and administered at 50 mg/kg by gavage using an esophageal cannula once daily throughout the study. The oral dosage was selected based on the previous literature that reported the hypoglycemic effects of naringenin in STZ-induced rodent models [4,7]. On the 4th day of oral administration, diabetes was induced by intraperitoneal injection of STZ dissolved in 50 mM citrate buffer (pH 4.5) at a dose of 50 mg/kg/day for four consecutive days. The control group received only the citrate buffer. All mice received a normal chow diet with water ad libitum during the experimental period. At the end of the study, mice were euthanized using an overdose of avertin (2,2,2-tribromoethanol). Blood was collected by cardiac puncture and centrifuged at 15,000× *g* at 4 °C for 20 min to collect serum samples. Pancreas, liver, and skeletal muscles were isolated for immunohistochemical staining and molecular analysis. All animal work was carried out in strict accordance with the institutional guidelines for the use and care of laboratory animals. The study protocol was approved by the Ethical Committee of Andong National University (Protocol Number: 2021-1-0128-01-01).

### 4.2. Measurements of Glucose, Insulin, and TBARS

Blood glucose levels in tail blood samples were measured every other day using a glucometer (OneTouch Ultra 2, LifeScan, Inc., Milpitas, CA, USA). The cumulative incidence of diabetes was calculated as the percentage of hyperglycemic mice (non-fasting blood glucose level ≥ 250 mg/dL) per treatment group at each time point. An oral glucose tolerance test (OGTT) was conducted on the 8th day after STZ injection was initiated. Briefly, after overnight fasting (16 h), mice were given glucose (2 g/kg body weight) by oral gavage. Blood glucose levels were monitored at the indicated time points before and after glucose administration. The area under the curve (AUC) for glucose during OGTT was calculated for each experimental group. Serum insulin levels were determined using an ELISA kit (Millipore Co., Billerica, MA, USA). Serum TBARS levels were determined by a colorimetric method as previously described [44].

### 4.3. Histology and Immunostaining

The isolated pancreas was fixed in 10% neutral buffered formalin, dehydrated using a series of graded alcohols, and embedded in paraffin. Sections (2 μm in thickness) were deparaffinized, rehydrated, and stained with hematoxylin and eosin (H&E). Four H&E-stained sections per mouse were used for measuring islet area using the ImageJ software (National Institutes of Health, NIH). For microscopy imaging of insulin, glucagon, and CD68, deparaffinized pancreas sections were incubated with primary antibodies (Abcam, Cambridge, UK) overnight at 4 °C. Staining was visualized using the mouse-specific HRP/DAB detection IHC kit (Abcam). Images of islets were obtaineded using a microscope (Leica Microsystems, Wetzlar, Germany). The percentage of target signal-positive area was calculated by dividing the area of target signal by the total islet area for at least 10 islets per mouse.

### 4.4. Western Blot Analysis

Pancreas, liver, and skeletal muscle tissues were homogenized in a lysis buffer containing a phenylmethylsulfonyl fluoride (Roche, Mannheim, Germany) and a protease inhibitor cocktail (Sigma-Aldrich, St. Louis, MO, USA) to prepare protein lysates. Total protein concentration was determined using the Bradford method. Equal amounts of protein were separated on 12% SDS/PAGE and transferred to polyvinylidene difluoride membranes. The membranes were blocked in PBS containing 3% BSA and 0.1% Tween-20 for 1 h at room temperature with constant agitation. These membranes were then probed with primary antibodies listed in Table S1, followed by incubation with corresponding horseradish peroxidase-conjugated secondary antibodies (Sigma-Aldrich). Protein bands were visualized using enhanced chemiluminescence reagents on a Fusion Solo 6S EDGE imaging system (Vilber, Marne-la-Vallée, France) and quantified using the ImageJ software.

### 4.5. Statistical Analysis

All statistical analyses were performed by one-way ANOVA using SAS software (SAS Institute Inc., Cary, NC, USA). For multiple comparisons among experimental groups, least squares means were used with Tukey–Kramer adjustments. Data are presented as means ± SEM. A value of *p* < 0.05 was considered statistically significant.

## Data Availability

The datasets used and/or analyzed during the current study are available from the corresponding author upon reasonable request.

## Supplementary Materials

The following supporting information can be downloaded at: https://www.mdpi.com/article/10.3390/molecules27134227/s1, Table S1: Antibodies used for Western blotting.
