# Supplementary material for: Naringenin and Phytoestrogen 8-Prenylnaringenin Protect against Islet Dysfunction and Inhibit Apoptotic Signaling in Insulin-Deficient Diabetic Mice
Source: Molecules. 2022 Jun 30;27(13):4227. doi: 10.3390/molecules27134227 (PMC9268740; doi:10.3390/molecules27134227)
Supplement: Supplementary file 1 [file molecules-27-04227-s001.zip › molecules-1780116-supplementary.pdf]

molecules

Article

Naringenin and Phytoestrogen 8-Prenylnaringenin Protect against Islet Dysfunction and Inhibit Apoptotic Signaling in Insulin-Deficient Diabetic Mice

Song Park [1], Kyu Sang Sim [2], Yeop Hwangbo [1], Sung Jin Park [3], Young Jun Kim [3] and Jun Ho Kim [1,*]

[1] Department of Food Science and Biotechnology, Andong National University, Andong 36729, Korea; andongori@naver.com (S.P.); qhduq97@naver.com (Y.H.)
[2] Biomaterials Research Institute, Kyochon F&B, Andong 36729, Korea; sim9612@naver.com
[3] Department of Food and Biotechnology, Korea University, Sejong 30019, Korea; timothypsj@hanmail.net (S.J.P.); yk46@korea.ac.kr (Y.J.K.)
* Correspondence: jhkim@anu.ac.kr; Tel.: +82-54-820-5846; Fax: +82-54-820-6264

Table S1. Antibodies used for western blotting.

Primary antibody	Clone	Company	Catalog No.	Dilution
**p65**	Polyclonal	Thermo Fisher	PA5-27617	1:1000
**p-p65**	Polyclonal	ABcam	ab86299	1:2000
**p-p53**	Polyclonal	ABcam	ab1431	1:1000
**p38**	Polyclonal	Cell Signaling	#9212	1:1000
**p-p38**	Polyclonal	ABcam	ab47363	1:2000
**JNK**	Monoclonal	ABcam	ab179461	1:2000
**p-JNK**	Monoclonal	ABcam	ab124956	1:2000
**ERK**	Polyclonal	ABcam	ab17942	1:2000
**p-ERK**	Monoclonal	ABcam	ab201015	1:2000
**p-p53**	Polyclonal	ABcam	ab1431	1:1000
**PI3K**	Monoclonal	Thermo Fisher	MA1-74183	1:1000
**p-PI3K**	Polyclonal	Thermo Fisher	PA5-104853	1:1000
**AKT**	Polyclonal	Cell Signaling	#9272	1:1000
**p-AKT**	Polyclonal	Cell Signaling	#9271	1:1000
**BAX**	Monoclonal	Cell Signaling	#2772	1:1000
**Bcl2**	Monoclonal	ABcam	ab692	1:500
**c-Cas3**	Polyclonal	Cell Signaling	#9661	1:1000
**c-Cas8**	Monoclonal	Cell Signaling	#8592	1:1000
**c-Cas9**	Polyclonal	Cell Signaling	#9509	1:1000
**c-PARP**	Monoclonal	ABcam	ab32064	1:1000
**TNF-α**	Polyclonal	ABcam	ab6671	1:2000
**IL-1β**	Polyclonal	Santa Cruz	sc-7884	1:1000
**ERα**	Monoclonal	Thermo Fisher	MA1-80216	1:1000
**ERβ**	Polyclonal	Thermo Fisher	PA1-310B	1:1000
**FGF21**	Monoclonal	Thermo Fisher	MA5-32652	1:1000
**GAPDH**	Monoclonal	Abbkine	A01020-SK	1:2000
